# Comparison of the effects of transversus thoracic muscle plane block and pecto-intercostal fascial block on postoperative opioid consumption in patients undergoing open cardiac surgery: a prospective randomized study

**DOI:** 10.1186/s12871-024-02432-w

**Published:** 2024-02-10

**Authors:** Mariana AbdElSayed Mansour, Hatem ElMoutaz Mahmoud, Dina Mahmoud Fakhry, Dina Yehia Kassim

**Affiliations:** Department of Anesthesiology, Surgical Intensive Care and Pain Management, Faculty of Medicine, Beni-Suef University, Beni-Suef, Egypt

**Keywords:** TTMPB, PIFB, Open cardiac surgery, Postoperative pain

## Abstract

**Background:**

There is an association exists between cardiac surgery, performed through median sternotomy, and a considerable postoperative pain.

**Objectives:**

The aim of the current study is to compare the effects of transversus thoracic muscle plane block (TTMPB) and pecto-intercostal fascial plane block (PIFB) upon postoperative opioid consumption among the patients who underwent open cardiac surgery.

**Methods:**

The present prospective, randomized, comparative study was conducted among 80 patients who underwent elective on-pump cardiac surgery with sternotomy. The subjects were randomly assigned to two groups with each group containing 40 individuals. For the TTMPB group, bilateral ultrasound-guided TTMPB was adopted in which 20 ml of 0.25% bupivacaine was used on each side. In case of PIFB group, bilateral ultrasound-guided PIFB was adopted with the application of 20 ml of 0.25% bupivacaine on each side. The researchers recorded the first time for rescue analgesia, the overall dosage of rescue analgesia administered in the first 24 h after the operation and the postoperative complications.

**Results:**

The PIFB group took significantly longer time to raise the first request for rescue analgesia (7.8 ± 1.7 h) than the TTMPB group (6.7 ± 1.4 h). Likewise, the PIFB group subjects had a remarkably lower ‘overall morphine usage’ in the first 24 h after the operation (4.8 ± 1.0 mg) than TTMPB group (7.8 ± 2.0 mg).

**Conclusion:**

Bilateral ultrasound-guided PIFB provided a longer time for the first analgesic demand than bilateral ultrasound-guided TTMPB in patients undergoing open cardiac surgery. In addition to this, the PIFB reported less postoperative morphine usage than the TTMPB and increases satisfaction in these patients.

**Trial registration:**

This study was registered at Clinical Trials.gov on 28/11/2022 (registration number: NCT05627869).

## Introduction

An association exists between cardiac surgery, performed through median sternotomy, and a considerable postoperative pain [1]. Sternotomy, sternal retraction, Internal Mammary Artery (IMA) harvesting, chest tubes, and steel materials cause pain to the patients after undergoing cardiac surgery [2]. The intercostal nerves transmit the feeling of pain in the sternum that originates from T2-T6 thoracic nerve roots. Post-sternotomy pain results in reduced patient satisfaction and other complications such as delirium, cardiovascular problems (e.g., hypertension, arrhythmias, and tachycardia), hyperglycemia, and respiratory problems (e.g., pneumonia, bronchial secretion stasis, and atelectasis) [3]. In literature, it has been reported that poor postoperative analgesia increases the morbidity rate and also lengthens the hospital stay among the patients, who underwent cardiac surgery compared to individuals who received appropriate postoperative analgesia [4]. Effective postoperative pain control is crucial to mitigate the risks involved in all the above-mentioned complications, including mortality and morbidity [5]. For patients who have undergone cardiac surgery, heavy dosage opioids can offer adequate postoperative analgesia. However, opioids have been established to have several adverse effects, which frequently delays the recovery process. Such adverse effects include nausea and vomiting, sedation, respiratory depression, ileus, cough suppression, lethargy, and delayed tracheal extubation [6]. Earlier, chest wall regional anesthesia was possible only through thoracic paravertebral blocks, thoracic epidural analgesia, and intercostal nerve blocks (ICNB). All these techniques are targeting the thoracic spinal nerves at or nearby their origin [7]. Despite efficacy, these strategies are rarely employed in patients who are undergoing cardiac surgery, due to the occurrence of adverse effects associated with potential epidural hematoma following full heparinization, sympathectomy-induced hypotension, pneumothorax, and spinal cord injury [8]. The emergence of the ultrasound-guided regional anesthesia resulted in the strategic development of fascial plane chest wall block. The transversus thoracic muscle plane block (TTMPB) is a recently-devised regional anesthesia method that provides analgesia to the anterior chest wall and was initially introduced by Ueshima et al. in 2015 [9]. In TTMPB, the local anesthetic (LA) is deeply administered at the fascial plane between the internal intercostal and transversus thoracic muscles. TTMPB covers the anterior branches of the intercostal nerves from T2 to T6 in order to effectively provide analgesia for the internal mammary region. Therefore, bilateral TTMPB is considered as an efficient analgesic replacement for those patients who are undergoing cardiac surgery [10]. A new, minimally-invasive technique called pecto-intercostal fascial plane block (PIFB) was firstly used by De la Torre in patients who had undergone breast surgery [11]. PIFB targets the anterior cutaneous branches of the intercostal nerves [12]. A few interfascial nerve block methods (for example, pectoral nerve blocks I and II and erector spinae plane block) are also utilized in some other procedures to reduce the postoperative pain. However, these methods require particular patient positioning. Some specific benefits have been reported when using PIFB such as less invasiveness, proximity to the incision line, and postoperative administration with no specific patient positioning [13, 14]. In this background, the current research article compared the impact of TTMPB and PIFB upon postoperative opioid usage among the patients who underwent open cardiac surgery.

## Methods

This prospective, randomized, and single-center study was conducted among 80 patients, who underwent elective on-pump cardiac surgery with sternotomy at Beni-Suef University Hospital, Beni Suef, Egypt, between February and August 2023. The present study received the approval from the Ethics Committee of the Faculty of Medicine, Beni-Suef University (code: FM-BSU REC/02102022/Mansour) and was registered at ClinicalTrials.gov (date: 28/11/2022; registration No.: NCT05627869). All the patients voluntarily participated in the study and written informed consent was obtained from all the patients. The current study also adhered to the Declaration of Helsinki’s principles. The study population comprised of 80 patients with the American Society of Anesthesiologists (ASA) grades I-III, female and male genders, age range of 18–75 years and had elective on-pump cardiac surgery with sternotomy. The exclusion criteria for the study are as follows; 1) emergent surgery, 2) off-pump surgery, 3) redo surgery, 4) ejection fraction less than 35%, 5) patient refusal, 6) hypersensitivity to LA, 7) chronic opioid usage or chronic pain patient, 8) psychiatric problems or communication difficulties, 9) liver insufficiency (serum bilirubin ≥ 34 μmol/l, albumin ≤ 35 g/dl, and international normalized ratio ≥ 1.7), 10) renal insufficiency (glomerular filtration rate < 44 ml/min), 11) obstructive sleep apnea syndrome, 12) coexisting hematologic disorders, and 13) pregnancy or breastfeeding.

The study subjects were randomly assigned to two groups with each group containing 40 individuals. For the TTMPB group, bilateral ultrasound-guided TTMPB was adopted using 20 ml of 0.25% bupivacaine (Sunnypivacaine®, Sunny Pharmaceutical) on each side. For the PIFB group, bilateral ultrasound-guided PIFB was adopted using 20 ml of 0.25% bupivacaine on each side. Computer-generated random numbers were assigned to the patients in order to ensure randomization. Subsequently, separate opaque envelopes were used to store the numbers under the supervision of a data administrator.

### Anesthetic procedure

Prior to the surgery, the study subjects were investigated in terms of complete blood count (CBC), coagulation profile, renal functions and electrolytes. As routine tests, electrocardiography, chest X-ray, and echocardiography were performed. A detailed medical history, including the medicines consumed, was taken on the night prior to the surgery. All the patients were explained about the study protocol, including the Visual Analog Scale (VAS), on the day of assessment before the operation. In the operating room, the heart rate, rhythm, and ST segments (leads II and V5) were monitored using a five-lead electrocardiogram (ECG) system. A pulse oximeter probe was attached to the patient while a peripheral venous cannula was placed.

The patients were anesthetized with propofol 0.5–1 mg/kg, midazolam 0.05–0.1 mg/kg, and fentanyl 2–5 µg/kg. Rocuronium 0.6–1.0 mg/kg was administered to ease orotracheal intubation using a cuffed tracheal tube. Anesthesia was maintained using 0.5–1.0% isoflurane in air and oxygen, intravenous infusion of fentanyl 2–5 μg/kg/hour and incremental doses of rocuronium 0.1–0.2 mg/kg every 30–45 min. All the patients were under mechanical ventilation in order to maintain normocarbia. After intravenous catheterization and endotracheal intubation, an arterial catheter was inserted into either right or the left radial artery for invasive arterial pressure monitoring and blood sampling. The central venous catheters were inserted through right internal jugular vein in order to monitor the central venous pressure and administer medication. Furthermore, an esophageal temperature probe and a urinary catheter were also placed. The coagulation profile of the patients was monitored based on activated clotting time. Complete cardiopulmonary bypass was started after sufficient heparinization and aortocaval cannulation were achieved. The activated clotting time reached a standard degree, following definitive surgery and protamine reversal of heparin.

### Intervention

All the blocks were administered after intubation, prior to the incision.

#### Ultrasound-guided TTMPB procedure

After placing the patients in supine position, a high-frequency convex array ultrasound transducer (PHILIPS HD5) was placed on one side of the sternum to capture the short axis views of partial sternum, internal intercostal muscle and transversus thoracic muscle. Based on the blood flow of internal mammary artery (IMA) and the internal mammary vein with color Doppler, the transversus thoracic muscle plane was identified. After iodine disinfection, a 22 G 80 mm needle (Pajunk SonoPlex® STIM; Geisingen, Germany) was inserted between the fourth and fifth ribs in-plane in the direction of cephalad to caudad [12]. The tip of the needle was put on the surface of transverse thoracic muscle plane between the ribs and above the pleura. Then, the study subjects were administered with 20 ml of 0.25% bupivacaine. The descending movement of pleura was utilized to determine the success of TTMPB. The technique was again adopted for the contralateral side.

#### Ultrasound-guided PIFB procedure

With the help of high-frequency linear ultrasound probe (PHILIPS HD5), PIFB was performed in supine position. The probe was put 2 cm laterally from the sternum and in parallel to it. Afterwards, the pectoralis major muscle, external intercostal muscle, costal cartilage, pleura and the lungs were detected. The location of the Pecto-Intercostal Fascial plane was found between pectoralis major muscle and external intercostal muscle or the costal cartilage. A 22 G 80 mm needle (Pajunk SonoPlex® STIM; Geisingen, Germany) was inserted under pectoralis major and above the external intercostal muscle following in-plane method. Furthermore, a test bolus of saline (2 mL) was injected to find out whether the tip is placed at the correct fascial layers. Eventually, 20 ml of 0.25% bupivacaine was injected into the plane at two locations, over the second rib and fourth rib. The same method was again adopted for the other side.

### Recovery and postoperative period

After the completion of cardiac surgery, all the subjects were transferred intubated to the intensive care unit (ICU) and their vitals such as hemodynamics, bleeding control, appropriate hemoglobin levels, serum electrolytes, and acid–base balance were maintained. Typical postoperative analgesia was performed through intravenous infusion of paracetamol (1 gm/6 h) and fentanyl (1 μg/kg/hour). Tracheal extubation was accomplished, in case if the subject fulfilled the criteria such as awake/arousable, hemodynamically stable, no active bleeding, warm peripheries, satisfactory arterial blood gas with a fraction of inspired oxygen < 0.5, decreased ventilator pressure support to 10 cm H_2_O, positive end-expiratory pressure 5–7 cm H_2_O, no electrolyte abnormalities and minimal or no escalation in inotropic support. Analgesia was continued for the patients using the same regimen. Supplemental rescue analgesia was provided in the form of intravenous morphine 0.05 mg/kg (at VAS ≥ 4). The present study defined the ‘postoperative first 24 h’ as the initial 24 h spent by the patients after the operation at the ICU following extubation. Subsequent to extubation, the study participants were assessed for the occurrence and severity of pain using the VAS scale at 0, 3, 6, 12, 18, and 24 h throughout breathing with standard tidal volume and coughing.

The following parameters were documented:

- Characteristic information: Age, gender, Body Mass Index (BMI), and ASA physical status
- Time required to adopt the procedure (minutes) (i.e., from placing the ultrasound probe on the patient’s skin to the termination of LA administration)
- Anesthesia duration and extubation time (i.e., since transmission to ICU to extubation)
- Heart rate (HR) and mean arterial blood pressure (MBP)
- The VAS scores for sternal pain at rest and with cough (range: 0–10 indicative of no and extreme pain, respectively) were recorded [15]. Pain relief is shown with a score of ≤ 3. Supplemental rescue analgesia i.e., morphine 0.05 mg/kg (at VAS ≥ 4) was intravenously provided.
- The first time for rescue analgesia (minute) is the time taken to raise a request for the initial postoperative analgesia (morphine) and was estimated from extubation to patient reporting a VAS score of ≥ 4.
- Overall dosage of rescue analgesia (morphine) (i.e., primary outcome) used in the initial 24 h after the surgery.
- The data regarding postoperative complications (e.g., bradycardia, hypotension, sedation, respiratory depression, nausea, and vomiting), throughout the initial 24 h after surgery, was collected. In order to evaluate nausea and vomiting, categorical scoring was employed with 0, 1, 2, and 3 indicating none, nausea, retching, and vomiting, respectively [16]. Sedation scale was applied to determine the sedation scores (0 = awake, 1 = drowsy, 2 = asleep but arousable, and 3 = deeply asleep). A sedation score of > 0 indicates being sedated at any time throughout the initial postoperative 24 h [17].
- Patient satisfaction was evaluated as follows; 1 = poor, 2 = moderate, 3 = good, and 4 = perfect [18].

The overall morphine usage is considered as the primary outcome (since the time from extubation to 24 h) whereas the first analgesic request time, the VAS score for pain during rest and with coughing, the time taken for extubation, and the side effects are the secondary outcomes.

### Sample size

The sample size was estimated by comparing the time taken to raise the first opioid request by the patients who underwent open cardiac surgery between TTMPB and PIFB. As reported in the literature [19], the median and interquartile range (IQR) of time for first opioid demand in the TTMPB group was approximately 240 (range: 161.3–525) minutes; however, in the PIFB group, it was approximately 660 (range: 540–900) minutes.

The present study considered the median to replace the mean and estimated the Standard Deviation (SD) from IQR by dividing it by 1.35 (Cochrane 2021). Accordingly, the minimum sample size for the current study was estimated to be 36 participants in each group, to provide the possibility of detecting an actual difference of 180 min with 80% power at 0.05 alpha by student’s t-test for the independent samples. The sample size was calculated by StatsDirect software (version 2.7.2). As a result, the study included 40 subjects in each group considering attrition of the samples.

### Statistical analysis

Data were statistically described in terms of mean ± standard deviation (± SD), median and range, or frequencies (number of cases) and percentages when appropriate. Because the groups are large enough, comparison of numerical variables between the study groups was done using Student t test for independent samples. Comparison of VAS over time within each group was done using Repeated measure ANOVA test. For comparing categorical data, Chi-square (χ^2^) test was performed. Exact test was used instead when the expected frequency is less than 5. Two-sided *p* values less than 0.05 was considered statistically significant. IBM SPSS (Statistical Package for the Social Science; IBM Corp, Armonk, NY, USA) release 22 for Microsoft Windows was used for all statistical analyses.

## Results

**Fig. 1:**
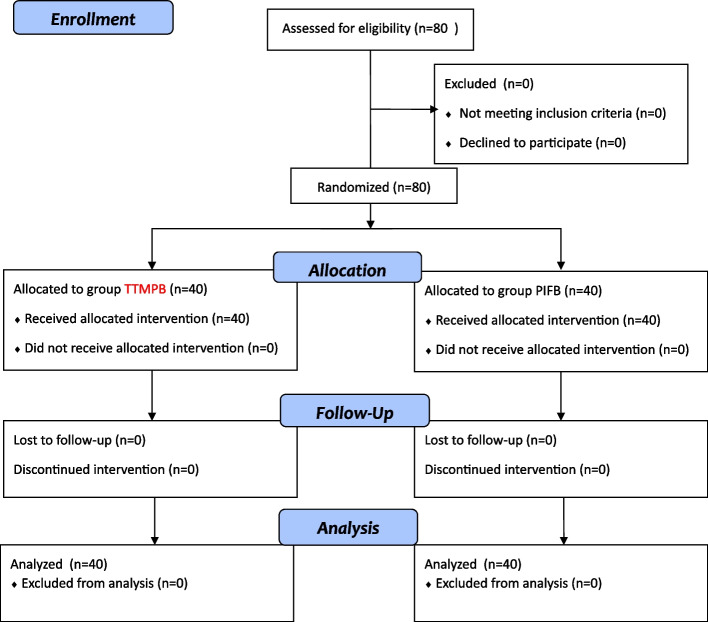
Flow diagram

**Fig. 2:**
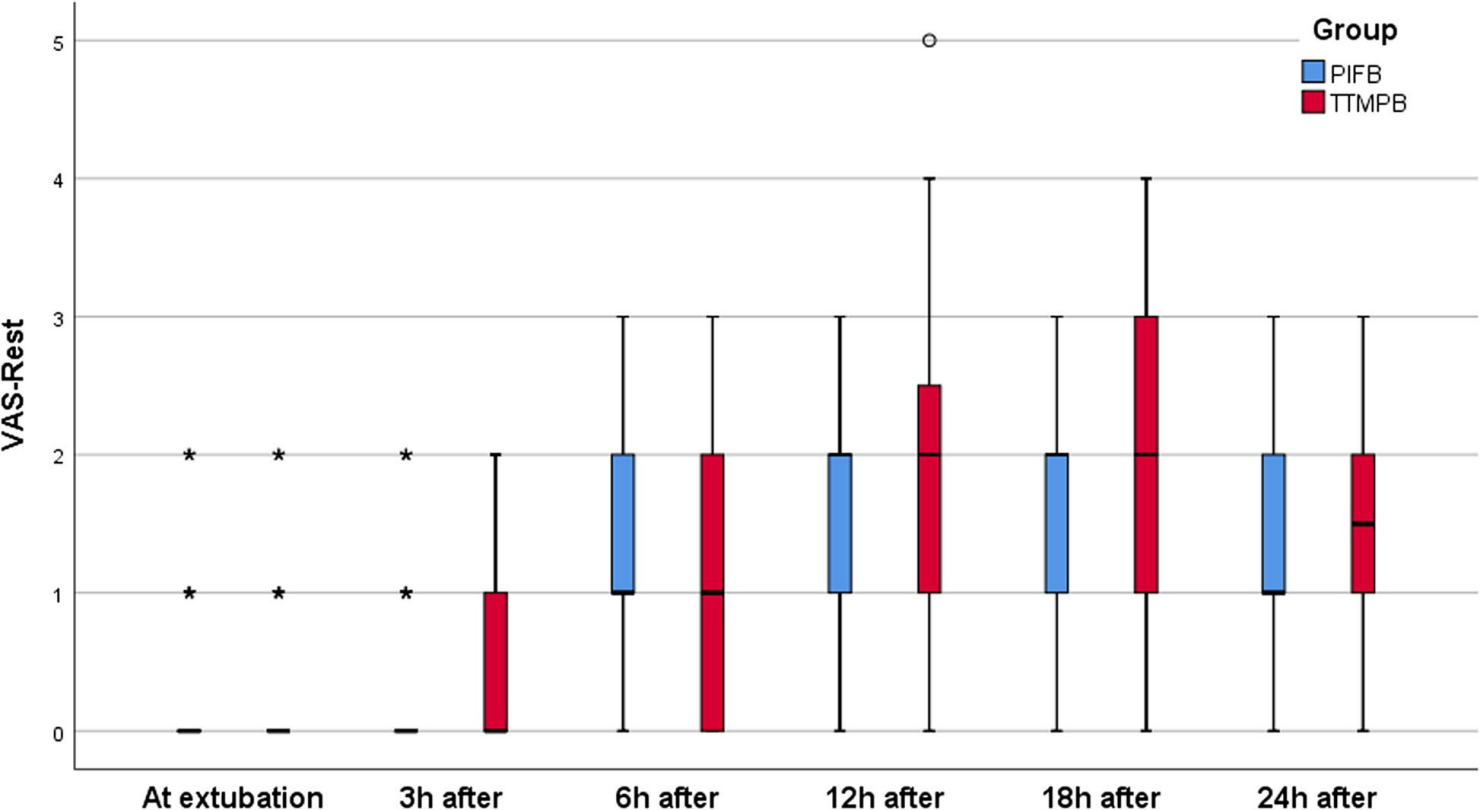
Median (range) VAS score at rest between the two groups over the study period

**Fig. 3:**
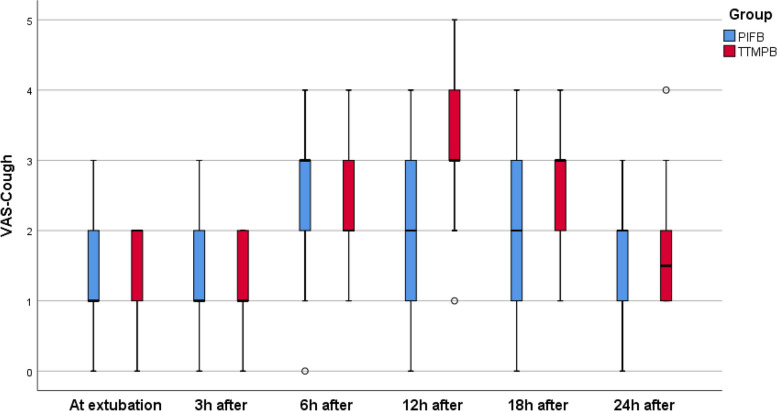
Median (range) VAS score during cough between the two groups over the study period

**Fig. 4:**
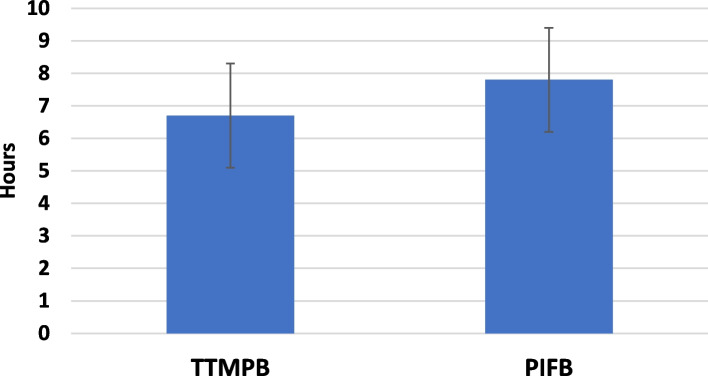
Mean (± SD) time to 1st analgesic request between the 2 study groups

**Fig. 5:**
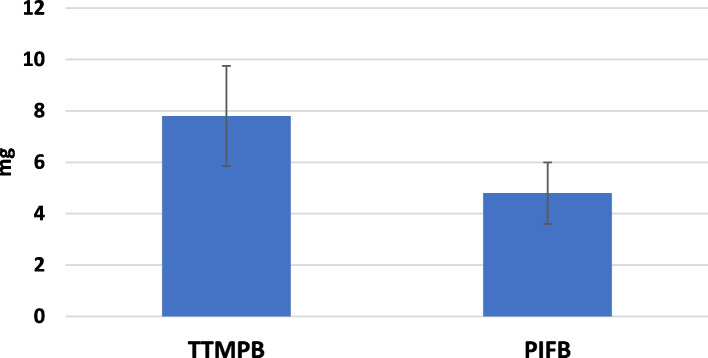
Mean (± SD) total morphine consumption between the 2 study groups

This study was carried out among 80 patients in two groups with each group containing 40 cases. None of the study subjects withdrew from the study (Fig. 1). Table 1 shows the demographic data of the patients. The study groups are comparable in terms of anesthesia duration, time required to use the technique and time for extubation (*P* > 0.05) (Table 2). According to the VAS scores calculated at rest during different time intervals, no statistical significant difference was observed between the groups at 0, 3, 6, 12, and 24 h after the surgery (*P* > 0.05). Nevertheless, there was a significant difference found in the VAS score at 18th hour after the surgery between the groups i.e., the PIFB group secured low VAS score than the TTMPB group (*P* < 0.05). In each group, the *p* value over time was < 0.001 (Table 3; Fig. 2). Additionally, the VAS scores determined during coughing showed no significant difference between the study groups at 0, 3, 6, and 24 h after the surgery (*P* > 0.05). Nonetheless, the PIFB group showed significantly low VAS scores during coughing at 12th and 18th hours after the surgery than the TTMPB group (*P* < 0.05) (Table 4; Fig. 3). In each group, *p* value over the time was < 0.001 (Table 4; Fig. 3). The time taken for raising the first request of rescue analgesic was significantly lengthier in the PIFB group (7.8 ± 1.7) than in case of TTMPB group (6.7 ± 1.4) (Table 5: Fig. 4). Likewise, the overall morphine usage, in terms of mg, during the first 24 h was remarkably lower in the PIFB group (4.8 ± 1.0) than the TTMPB group (7.8 ± 2.0) (Table 5; Fig. 5). Regarding MAP, there was no significant difference between both groups or over time in each group. Also, there was no significant difference in HR between the two groups or over time in each group. No significant difference was found between the groups in terms of frequency of cases with nausea and vomiting (*P* > 0.05). With regards to sedation scores, no remarkable difference was observed between the groups (*P* > 0.05). Additionally, there was no significant difference found between the groups according to patient satisfaction scores (*P* > 0.05) (Table 6).

**Table 1:** Demographic data between the two groups

	**TTMPB (*n* = 40)**	**PIFB (*n* = 40)**	***p* value**
Age (years)	53.3 ± 15.2	52.7 ± 15.8	0.880
Males/Females	28/12	29/11	0.805
BMI (kg/m^2^)	30.1 ± 6.7	34.3 ± 8.6	0.017
ASA I/ II/ III	7/23/10	0/29/11	0.021

Data are presented as mean ± standard deviation (SD) or as the number of patients

*P*-value < 0.05 (significant), *P*-value > 0.05 (non-significant)

**Table 2:** Time needed to perform technique, Anesthesia duration, time to extubation between the two groups

	**TTMPB (*n* = 40)**	**PIFB (*n* = 40)**	***p* value**
Time needed to perform technique (min)	4.2 ± 0.8	4.1 ± 0.8	0.490
Anesthesia duration (min)	286.4 ± 33.5	279.1 ± 40.4	0.380
Time to extubation (min)	250.0 ± 64.0	250.5 ± 82.4	0.976

Data are presented as mean ± standard deviation (SD)

*P*-value < 0.05 (significant), *P*-value > 0.05 (non-significant)

**Table 3:** VAS score between the two groups at 0, 3, 6, 12, 18, 24 h at rest

	**TTMPB (*n* = 40)**	**PIFB (*n* = 40)**	***p* value**
VAS at extubation	0 (0–2)	0 (0–2)	0.859
VAS-3h after extubation	0 (0–2)	0 (0–2)	0.601
VAS-6h after extubation	1 (0–3)	1 (0–3)	0.210
VAS-12h after extubation	2 (0–5)	2 (0–3)	0.069
VAS-18h after extubation	2 (0–4)	2 (0–3)	0.004
VAS-24h after extubation	1.5 (0–3)	1 (0–3)	0.106
*P* value (over time)	< 0.001	< 0.001	

Data are presented as median (range)

*P*-value < 0.05 (significant), *P*-value > 0.05 (non-significant)

**Table 4:** VAS score between the two groups during cough at 0, 3, 6, 12, 18, 24 h

	**TTMPB (*n* = 40)**	**PIFB (*n* = 40)**	***p* value**
VAS at extubation	2 (0–2)	1 (0–3)	0.252
VAS-3h after extubation	1 (0–2)	1 (0–3)	0.527
VAS-6h after extubation	2 (1–4)	3 (0–4)	0.388
VAS-12h after extubation	3 (1–5)	2 (0–4)	< 0.001
VAS-18h after extubation	3 (1–4)	2 (0–4)	< 0.001
VAS-24h after extubation	1.5 (1–4)	2 (0–3)	1.000
*P* value (over time)	< 0.001	< 0.001	

Data are presented as median (range)

*P*-value < 0.05 (significant), *P*-value > 0.05 (non-significant)

**Table 5:** The time to first request of rescue analgesic and total morphine consumption between the two groups

	**TTMPB (*n* = 40)**	**PIFB (*n* = 40)**	***p* value**
Time to first request of rescue analgesic (h)	6.7 ± 1.4	7.8 ± 1.7	0.003
Total morphine-24h (mg)	7.8 ± 2.0	4.8 ± 1.0	< 0.001

Data are presented as mean ± standard deviation (SD)

*P*-value < 0.05 (significant), *P*-value > 0.05 (non-significant)

**Table 6:** Nausea and vomiting, sedation score, patients’ satisfaction between the two groups

	**TTMPB (*n* = 40)**	**PIFB (*n* = 40)**	***p* value**
Nausea & Vomiting:			
- None	32 (80%)	36 (90%)	0.335
- Nausea	4 (10%)	2 (5%)	
- Retching	4 (10%)	2 (5%)	
- Vomiting	0 (0%)	0 (0%)	
Sedation:			
- Awake	32 (80%)	32 (80%)	0.539
- Drowsy	5 (13%)	4 (10%)	
- Asleep but arousable	2 (5%)	2 (5%)	
- Deep sleep	1 (3%)	2 (5%)	
Satisfaction			
- Poor	3 (8%)	2 (5%)	0.373
- Moderate	8 (20%)	3 (8%)	
- Good	16 (40%)	18 (45%)	
- Perfect	13 (33%)	17 (43%)	

Data are presented as numbers and percent

*P*-value < 0.05 (significant), *P*-value > 0.05 (non-significant)

## Discussion

Patients undergoing open cardiac surgery typically suffer from long duration and serious postoperative pain for which multimodal analgesia should be under control. The new advanced chest wall regional block technique offers effective postoperative analgesia by providing enhanced recovery after cardiac surgery, thereby reducing the opioid requirement and the complications associated with it.

The current randomized controlled study compared the effects of bilateral ultrasound-guided TTMPB and bilateral ultrasound-guided PIFB upon the postoperative sternotomy pain induced by cardiac surgery. The results confirmed that the time taken to request for initial analgesic got prolonged in the PIFB group than in the TTMPB group. Additionally, the postoperative morphine usage was found to be lower in PIFB than the TTMPB group. However, no remarkable difference was found between the groups in terms of VAS scores at various time intervals at rest, except at the 18th hour after the surgery. The PIFB group patients secured low VAS scores. Similarly, the VAS score during coughing was comparable between the groups except at the 12th and 18th hours after the surgery. In this scenario, the PIFB group secured low scores than the TTMPB group. Both the groups were compared regarding the frequency of subjects with postoperative nausea and vomiting, sedation scores, and patient satisfaction scores.

According to the evidence, the pain occurs after sternotomy through T2-T6 intercostal nerves; therefore, blocking the aforementioned nerves can effectively alleviate the sternotomy-induced pain and reduce the duration of ICU and hospital stay [20]. Various investigations established the successful adoption of TTMPB for pain control following sternotomy in both adult and pediatric populations who are undergoing cardiac surgery. Kendigelen et al. [21] reported the TTMPB length to be above 48 h, because it is one of the interfascial blocks that has been recognized for prolonged analgesia. Fujii et al. [22] investigated the effectiveness of single-shot TTMPB among adults who underwent elective cardiac surgery and the study demonstrated remarkably low pain scores among the patients with TTMPB than the controls. Nonetheless, 24-h overall hydromorphine doses were the same in both the groups, which might be attributed to several issues. Firstly, the aforementioned investigation was a pilot study and recruited only 19 subjects due to which the results are too narrow to declare a difference. Secondly, there was no control over intraoperative and ICU opioid usage that tend to affect the postoperative pain scores and opioid demands. Moreover, 60% of the aforementioned study subjects underwent Coronary Artery Bypass Grafting (CABG) with IMA harvesting. IMA harvesting leads to surgical disturbance of TTMPB and uneven spread of the injectate between the intended thoracic levels. As a result, the patients might not gain the advantages from TTMPB on the mentioned side [23].

Additionally, Muhammed Enes Aydin et al. [24] conducted a study on the effectiveness of ultrasound-guided TTMPB on opioid usage following cardiac surgery and concluded that a single preoperative TTMPB allowed effective analgesia and reduced the requests for opioids from the cardiac surgery patients. Abd Elbaser et al. [25] adopted TTMPB among the pediatric subjects who underwent cardiac surgery through median sternotomy. The TTMPB group demonstrated remarkably low fentanyl demands after the surgery than the saline group. Furthermore, the TTMPB group also recorded significantly low pain scores during all the time intervals. No significant complications, including pneumothorax, hematoma, and hemothorax, were observed through a retrospective review of TTMPB techniques among 299 adults [26].

In 2014, de la Torre et al. [11] illustrated the ultrasound-guided PIFB in which the injection is done at 2 cm lateral to the sternum and between the pectoralis major and the external intercostal muscles in breast surgery. They concluded that the aforementioned procedures are highly advantageous than ICNB. These methods reduce the dosage of LA, frequency of required punctures and the minimization of unexpected and unwanted pleural and vessel punctures. In addition to these, these novel methods probably reduce LA systemic absorption than the ICNB process and can be easily adopted. According to de la Torre et al.’s experience, this method is safe and efficient with good analgesic quality following breast surgery. PIFB results in efficacious analgesia in case of breast surgery [12], sternal fracture pain [27], rib cage discomfort in ICU patients [28], and the implantation of subcutaneous-implantable cardioverter defibrillator system [29]. Additionally, some studies demonstrated the application of PIFB for thymectomy through median sternotomy [30, 31] and cardiac surgery [32]. A few case reports have drawn particular attention to the significance of PIFB in reducing the opiate usage & pain scores and also enhancing the patient satisfaction outcomes among the post-sternotomy patients [33]. According to a case report by Victor et al. [32], PIFB can be successfully adopted to treat a patient after CABG with retractable pain that is generally not relieved by opioids or other analgesics. The PIFB can be adopted using a catheter-based strategy with a great success for pain control in sternal fractures [27]. Mohamed A Hamed et al. [34] investigated the analgesic impact of ultrasound-guided bilateral PIFB upon sternal wound pain following open cardiac surgery and demonstrated that the increasing morphine usage, during the initial 24 h, got remarkably lower in the block group. Furthermore, the median estimated time for the first analgesic demand got significantly prolonged in the block group than the control group. Patients who underwent CABG with IMA harvesting protocol suffered from more pain. The PIFB, performed in proximity to the IMA dissection site, provided higher pain relief and recovery after the surgery [35]. Numerous functions have been reported for PIFB such as the perioperative pain control in cardiac surgery, the only anesthetic for breast surgery, and analgesia for rib and sternal fractures [28, 36]. Cengiz Kaya et al. [19] compared the effects of PIFB and TTMPB and concluded that the time taken for first rescue analgesia demand got prolonged in PIFB group without a significant difference between the groups in terms of morphine usage after 24 h of operation. There exists a few solid reasons to consider PIFB as a substitute for TTMPB [22, 37]. The first reason is that the transversus thoracic muscle is usually very thin, thus making it a challenge to visualize under ultrasound, and in close proximity to the pleura [38]. This issue increases the risk of pneumothorax in TTMPB. The second reason is that the IMA and vein pass through the interfascial plane. So, when performing the block, the needle point will be on this plane. As a result, the risk of vascular laceration in TTMPB is high. The third reason is that tissue disruption is possible during TTMPB in CABG, when the artery harvest affects the LA spread [23]. So, PIFB remains the best option for these cases in open cardiac surgery [39]. The current study has a few limitations that have to be overcome in future studies. Firstly, the sample size was modest, thereby requiring further clinical studies with a larger population. Secondly, it was impossible to carry out a dermatomal examination due to the administration of the blocks following anesthesia induction. Thirdly, the patients were followed up for pain scores only for 24 h. Fourthly, the study lacked a no-block control group. Finally, the restricted number of accessible clinical studies made the comparison difficult.

## Conclusion

Bilateral ultrasound-guided PIFB provided a longer time for the first analgesic demand than bilateral ultrasound-guided TTMPB in patients undergoing open cardiac surgery. In addition to this, the PIFB reported less postoperative morphine usage than the TTMPB and increases satisfaction in these patients. Both the techniques were compared regarding the frequency of subjects with postoperative nausea and vomiting, sedation scores, and patient satisfaction scores.

## Data Availability

The datasets used and analysed during the current study are available from the corresponding author upon reasonable request.
